# Vitamin A Status and Deposition in Neonatal and Weanling Rats Reared by Mothers Consuming Normal and High-Fat Diets with Adequate or Supplemented Vitamin A

**DOI:** 10.3390/nu12051460

**Published:** 2020-05-18

**Authors:** Yanqi Zhang, Kristi M. Crowe-White, Lingyan Kong, Libo Tan

**Affiliations:** Department of Human Nutrition, University of Alabama, Tuscaloosa, AL 35487, USA; yzhang309@crimson.ua.edu (Y.Z.); kcrowe@ches.ua.edu (K.M.C.-W.); lkong@ches.ua.edu (L.K.)

**Keywords:** retinol, retinyl esters, maternal obesity, maternal supplementation, adipose tissue, neonate, neonatal obesity

## Abstract

The circulating level of vitamin A (VA; retinol) was reported to be lower in obese adults. It is unknown if maternal obesity influences the VA status of offspring. The objective of the study was to determine the VA status and deposition of neonatal and weanling rats reared by mothers consuming a normal or high-fat diet (NFD or HFD) with or without supplemented VA. Pregnant Sprague-Dawley rats were randomized to an NFD or HFD with 2.6 mg/kg VA. Upon delivery, half of the rat mothers in the NFD or HFD cohort were switched to an NFD or HFD with supplemented VA at 129 mg/kg (NFD+VA and HFD+VA group). The other half remained on their original diet (NFD and HFD group). At postnatal day 14 (P14), P25, and P35, pups (n = 4 or 3/group/time) were euthanized. The total retinol concentration in the serum, liver, visceral white adipose tissue (WAT), and brown adipose tissue (BAT) was measured. At P14, the HFD+VA group showed a significantly lower serum VA than the NFD+VA group. At P25, both the VA concentration and total mass in the liver, WAT, and BAT were significantly higher in the HFD+VA than the NFD+VA group. At P35, the HFD group exhibited a significantly higher VA concentration and mass in the liver and BAT compared with the NFD group. In conclusion, maternal HFD consumption resulted in more VA accumulation in storage organs in neonatal and/or weanling rats, which potentially compromised the availability of VA in circulation, especially under the VA-supplemented condition.

## 1. Introduction

Vitamin A (VA, retinol) is an essential nutrient required for the development of newborns (birth to 1 month) and infants (1 month to 2 years), in terms of growth, hematopoiesis, lung maturation, and immunity [1]. VA-deficient infants are at a higher risk of mortality and infectious diseases [2]. VA is also critical for the postnatal development of the lung [3]. For example, rapid utilization of the storage form of VA, i.e., retinyl esters, and of the production of retinoic acid, the active metabolite of VA, in newborn rats during lung development was reported [4]. In spite of the significance of VA for infants, they are born with low stores of VA, partly due to limited placental transfer. Thus, they rely on breast milk to build up this crucial nutrient [5]. VA levels in the serum, liver, and extrahepatic tissues in healthy newborns were reported to be lower compared with those in adults [5,6,7,8]. VA levels are even lower in low birth weight or preterm newborns [9,10]. As such, maternal VA intake is critical to establishing an optimal VA status during infancy.

In the U.S., although less than 1% of the population is reported to be deficient in VA based on the serum retinol concentration [11], dietary surveys indicated that 51% of adults, including women of childbearing age, do not meet the estimated average requirement (EAR) of VA, the dietary reference intake (DRI) value that is used to assess the nutrient intake of an individual or of a group [12]. Moreover, it should be noted that DRIs were developed for the healthy general population to ensure their optimal nutritional status. However, considering the rising rate of overweight and obesity in reproductive-aged women, which has achieved 48% in the U.S. [13], this question should be asked: Would maternal obesity, and increased adiposity in offspring that usually follows [14], affect VA metabolism and compromise the VA status in newborns and infants? If the answer is yes, it means overweight and obese pregnant or lactating women may require more VA than the current dietary recommendation to ensure the VA status and normal development of their children.

As a fat-soluble vitamin, VA accumulates in fatty tissues, i.e., the liver and adipose tissue, for long-term storage. One theory is that enlarged adipose tissue or liver as seen in overweight/obese individuals would act as a “sink” and sequester more VA, resulting in less in circulation or delivery to target organs for functions. Previous studies indicated that serum retinol is negatively correlated with body weight in morbidly obese adults [15,16], the population in which VA deficiency and night blindness were found to be highly prevalent [17]. Serum VA status was also reported to be significantly lower in obese school-aged children than in normal weight children [18]. Using obese mice, generated from the consumption of a high-fat diet or by genetic mutations, Trasino et al. observed an elevated serum retinol level but greatly reduced VA levels in multiple organs, including the liver, lung, pancreas, and kidney, even though these mice received adequate VA from their diet [19]. Additionally, it was reported that the ratio of serum retinol to retinol-binding protein (RBP), the transport protein for retinol in circulation, was significantly lower in obese than in nonobese people due to an elevated apo-RBP concentration [16], indicating the possibility that the mobilization of VA from the liver in obese individuals may be impaired. Despite these previous findings, to date, the impact of maternal obesity on the VA status in offspring has never been studied.

From another point of view, VA was shown to be a key regulator of adipose tissue development [20,21,22]. Using adult obese rodent models, it was shown that chronic dietary VA supplementation reduced the body weight gain, adiposity, and white adipose tissue (WAT) mass [23,24], while retinoic acid was shown to regulate adipogenesis [25,26], angiogenesis, and apoptosis [27] of WAT and the adaptive thermogenesis of brown adipose tissue (BAT) [28]. A previous study conducted in mice indicated that maternal dietary VA supplementation reduced the adiposity of offspring challenged with a high-fat diet [29]. Maternal VA supplementation, which was also reported to have a significantly greater sustained effect on improving the neonatal VA status compared to VA treatment directly administered to neonates [30], was therefore proposed to be a potential strategy in managing overweight or obesity in early life.

Based on these previous findings, the objective of this exploratory study was to determine the status and deposition of VA in neonatal and weanling Sprague-Dawley rats reared by mothers consuming normal and high fat diets with adequate or supplemented VA. It was hypothesized that maternal high fat diet-induced obesity would lead to a significantly higher concentration or total mass of VA in the storage organs of rat pups and a lower level in circulation under both VA-adequate and -supplemented conditions, which potentially compromises the availability of VA for utilization.

## 2. Materials and Methods

### 2.1. Animal Experiment

Pregnant Sprague-Dawley rats were purchased from Charles River Laboratories (Wilmington, MA, USA) and arrived at their second day of gestation. Rats were housed individually in solid cages under normal 12-h/light/dark cycle in a climate- and humidity-controlled environment at the University of Alabama Animal Care Facility. After a 3-day acclimation, rats were randomized to either a normal-fat diet (NFD; 25% fat) or a high-fat diet (HFD; 50% fat) both containing VA in the form of retinyl acetate at 2.6 mg/kg, a level that can maintain a healthy pregnancy and normal fetal development. Upon the delivery of rat pups, half of the rat mothers in the NFD or HFD cohort were switched to an NFD or an HFD with supplemented VA at 129 mg/kg (designated as the NFD+VA and HFD+VA group, respectively). The other half remained on their diets with 2.6 mg/kg of VA (designated as the NFD and HFD group, respectively) through lactation.

At postnatal day 14 (P14) and P25, 4 pups/group were euthanized. At P25, the remaining weanling pups (n = 3/group) were fed the diets of their respective mother until being euthanized at P35. At each sacrifice day, rat pups were euthanized via carbon dioxide, blood was collected from the vena cava, and tissues, including visceral WAT, BAT, and liver, were collected. At the P14 sacrifice, the stomach was also collected to obtain milk samples. Blood samples were centrifuged to obtain serum. Serum and tissues were stored at −80 °C until analysis. The animal study protocol was approved by the Institutional Animal Care and Use Committee at the University of Alabama (Protocol #18-05-1166; approved on 28 September 2018).

### 2.2. Analysis of Total Retinol

The concentration of total retinol (esterified + unesterified retinol) in serum, milk separated from the stomach, liver, WAT, and BAT, was analyzed by ultra-high-performance liquid chromatography (UPLC) (Acquity UPLC System; Waters, Milford, MA, USA) using an adaptation of previously reported methods [31,32]. For serum, 100 µL of the sample was incubated with 1.9 mL of 100% ethanol at room temperature for one hour for lipid extraction. For milk residue, liver, WAT, and BAT, 0.1 g of tissue was cut, homogenized, and incubated with 1.9 mL of 100% ethanol at room temperature for an hour. An amount of 100 µL of 100% potassium hydroxide and 100 µL of 20% pyrogallol were added to the sample and incubated in a water bath at 55 °C for saponification. After 30 min, 4 mL of hexane (with 0.1% butylated hydroxytoluene) and 2 mL of dd H_2_O were added. After centrifuging, the upper phase was collected and dried under nitrogen. The dried sample was then rinsed with hexane twice, dried, and reconstituted with 100 µL of acetonitrile:methanol (85:15, *v*/*v*). The reconstituted sample was then centrifuged for 2 min to remove any possible precipitation. For the UPLC analysis, 10 µL of sample was injected onto the Waters Acquity UPLC HSS T3 (1.8 μm, 2.1 mm × 100 mm) column (Waters, Milford, MA, USA). Retinyl acetate (Sigma-Aldrich, St. Louis, MO, USA) was used as the internal standard.

### 2.3. Statistical Analysis

All data are reported as mean ± standard error of mean (SEM). Body weight and tissue weight at individual time points were analyzed by one-way ANOVA followed by Bonferroni post test. The serum and tissue total retinol concentrations and mass were compared between different groups at individual time points using Student’s *t* test. All data analysis was conducted using GraphPad Prism software (San Diego, CA, USA). *p* < 0.05 was considered as statistically significant.

## 3. Results

### 3.1. Dietary Intake, Body Weight, and Organ Weights

No difference in the daily food intake was observed among groups during the suckling or postweaning period. The body weight and weights of the liver, visceral WAT, and BAT of rat pups are shown in Table 1.

At P14 and P25, pups in the HFD group had a significantly increased body weight and WAT mass as compared to the NFD group (*p* < 0.001), confirming the effectiveness of maternal HFD consumption. No significant difference in body weight or WAT mass was noted between the NFD and NFD+VA group, while the HFD+VA group showed a significantly lowered body weight (*p* < 0.05) and WAT mass (*p* < 0.01) compared to the HFD group. At P25, the BAT mass was significantly lower in the HFD group compared with the NFD group (*p* < 0.05), while maternal VA supplementation significantly increased the mass (*p* < 0.05). At P25, the liver weight of pups in the HFD and HFD+VA groups was significantly higher than that in the NFD and NFD+VA groups (*p* < 0.05), while VA supplementation exerted no significant effect. At P35, the time point in the postweaning period, HFD+VA pups still had a significantly lower WAT mass compared to the HFD group (*p* < 0.05). No significant difference in body weight, BAT weight, or liver weight was observed among the groups.

### 3.2. Serum and Milk Total Retinol Concentration

**Figure 1:**
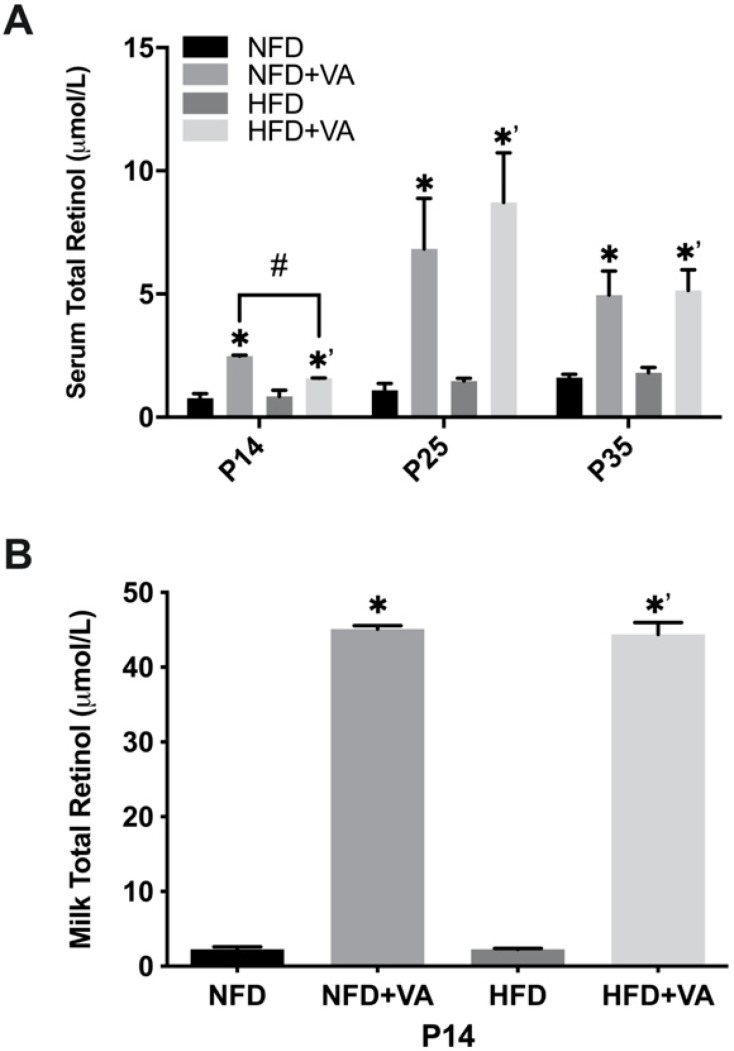
Concentrations of total retinol in the serum (**A**) of rat pups and in milk (**B**). Bars show mean ± SEM, *n* = 4 or 3 per group. Significant difference, *p* < 0.05, between NFD and NFD+VA was indicated by *, between HFD and HFD+VA by *^’^, and between NFD and HFD or between NFD+VA and HFD+VA by #. P14, postnatal day 14; P25, postnatal day 25; P35, postnatal day 35; NFD, normal fat diet; HFD, high fat diet; VA, vitamin A.

As shown in Figure 1, at all three time points, the VA-supplemented groups showed a significantly higher serum total retinol concentration as compared to their relative control group (*p* < 0.01). At P14, the serum retinol of pups in the NFD and HFD group was 0.79 ± 0.32 and 0.84 ± 0.43 µmol/L, respectively, corresponding to a VA marginal (serum retinol: 0.35–0.7 µmol/L) to normal status (serum retinol: 0.7–1.75 µmol/L) [33]. Maternal VA supplementation significantly raised the concentration to a normal or even elevated (serum retinol > 1.75 µmol/L) status at P14 [34], achieving 2.48 ± 0.04 and 1.57 ± 0.02 µmol/L in the NFD+VA and HFD+VA group, respectively. At P25 and P35, the serum retinol of NFD and HFD pups rose to the normal status, with a range from 1.09 to 1.8 µmol/L. The serum retinol concentration of NFD+VA and HFD+VA pups ranged from 6.83 to 8.71 µmol/L at P25, and from 4.95 to 5.13 µmol/L at P35, both corresponding to an elevated status.

Comparing the serum retinol concentration between the NFD and HFD group at three time points, no significant difference was noted. However, at P14, a significantly lower serum retinol concentration was observed in the HFD+VA group compared to the NFD+VA group (*p* < 0.05). No such difference was noted at P25 or P35.

For the milk VA concentration, the VA-supplemented groups showed significantly higher concentrations compared to their respective controls (*p* < 0.05); no significant difference was noted between the NFD and HFD group or between the NFD+VA and HFD+VA group.

### 3.3. Liver Total Retinol Concentration and Mass

**Figure 2:**
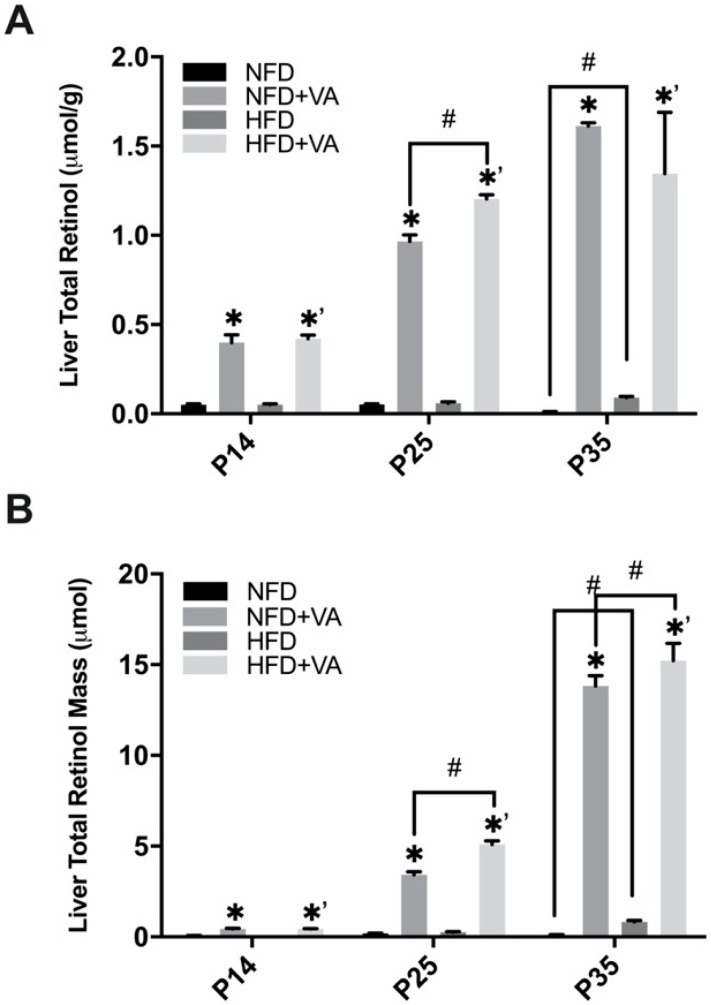
Liver total retinol concentration (**A**) and mass (**B**) of rat pups. Bars show mean ± SEM, *n* = 4 or 3 per group. Significant difference, *p* < 0.05, between NFD and NFD+VA was indicated by *, between HFD and HFD+VA by *^’^, and between NFD and HFD or between NFD+VA and HFD+VA by #. P14, postnatal day 14; P25, postnatal day 25; P35, postnatal day 35; NFD, normal fat diet; HFD, high fat diet; VA, vitamin A.

Through the period of P14 to P35, the liver total retinol concentration of pups in non-supplemented groups, i.e., the NFD and HFD group, ranged from 0.011 to 0.06 µmol/g (Figure 2A), which falls into a VA-deficient (liver VA store < 0.035 µmol/g) to marginal status (liver VA store: 0.035–0.07 µmol/g) [34]. At all three time points, the hepatic total retinol concentrations were significantly increased in VA-supplemented groups as compared to their respective control (*p* < 0.05). At P14, P25, and P35, pups in the supplemented groups exhibited a liver total retinol of 0.31–0.49 µmol/g, 0.89–1.25 µmol/g, and 1.15–1.65 µmol/g, respectively, all corresponding to a VA adequate (liver VA store > 0.07 µmol/g) or even sub-toxic status (liver VA store > 1 µmol/g) [35].

At P14 and P25, comparison between the NFD and the HFD group showed no significant difference in the liver total retinol concentration (Figure 2A) or mass (Figure 2B; concentration x organ weight). However, at P35, pups in the HFD group showed a significantly higher hepatic total retinol concentration and mass compared with the NFD group (*p* < 0.05). Comparing the NFD+VA and HFD+VA group, at P25, both the liver total retinol concentration and mass were significantly higher in the HFD+VA group (*p* < 0.05). In addition, at P35, the HFD +VA group also possessed a significantly higher VA mass than the NFD+VA group (*p* < 0.05).

### 3.4. Adipose Tissues Total Retinol Concentration and Mass

**Figure 3:**
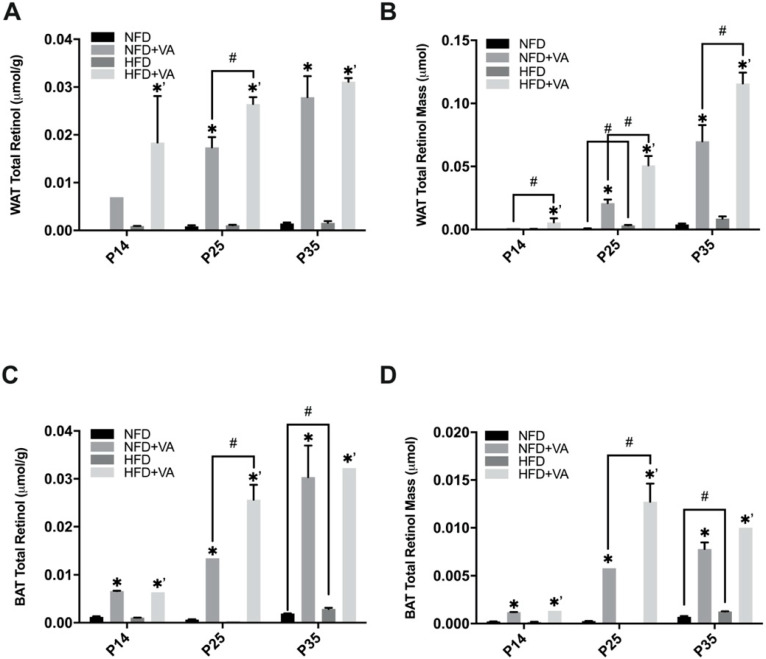
Total retinol concentration and mass of white adipose tissue (**A**,**B**) and brown adipose tissue (**C,D**). Bars show mean ± SEM, *n* = 4 or 3 per group. Significant difference, *p* < 0.05, between NFD and NFD+VA was indicated by *, between HFD and HFD+VA by *^’^, and between NFD and HFD or between NFD+VA and HFD+VA by #. P14, postnatal day 14; P25, postnatal day 25; P35, postnatal day 35; NFD, normal fat diet; HFD, high fat diet; VA, vitamin A. Note: At P14, white adipose tissue samples in the NFD group were not adequate for analysis, and therefore data are missing.

As shown in Figure 3, VA supplementation with either the NFD or the HFD significantly increased the WAT total retinol concentration (Figure 3A) and mass (Figure 3B) in pups (*p* < 0.05). At P25, both the concentration and the mass of total retinol in WAT was significantly higher in the HFD+VA than in the NFD+VA group (*p* < 0.05). The difference in total retinol mass between these two groups was also observed at P35. Additionally, at P25, the total retinol mass of WAT was significantly higher in the HFD group compared to the NFD group (*p* < 0.001). No significant difference in the VA concentration or mass in WAT was found between the NFD and the HFD group at P35. WAT collected from the NFD group at P14 was not adequate for analysis, so data was missing.

As for the BAT, the VA-supplemented groups showed significantly higher concentrations (Figure 3C) and mass (Figure 3D) of total retinol compared with their respective controls (*p* < 0.01). At P25, a significantly higher retinol concentration and mass were noted in the HFD+VA group as compared to the NFD+VA group (*p* < 0.05). At P35, pups in the HFD group showed a significantly increased VA concentration and mass than the NFD group (*p* < 0.05).

### 3.5. VA Distribution in Primary Storage Sites Versus in Circulation

**Figure 4:**
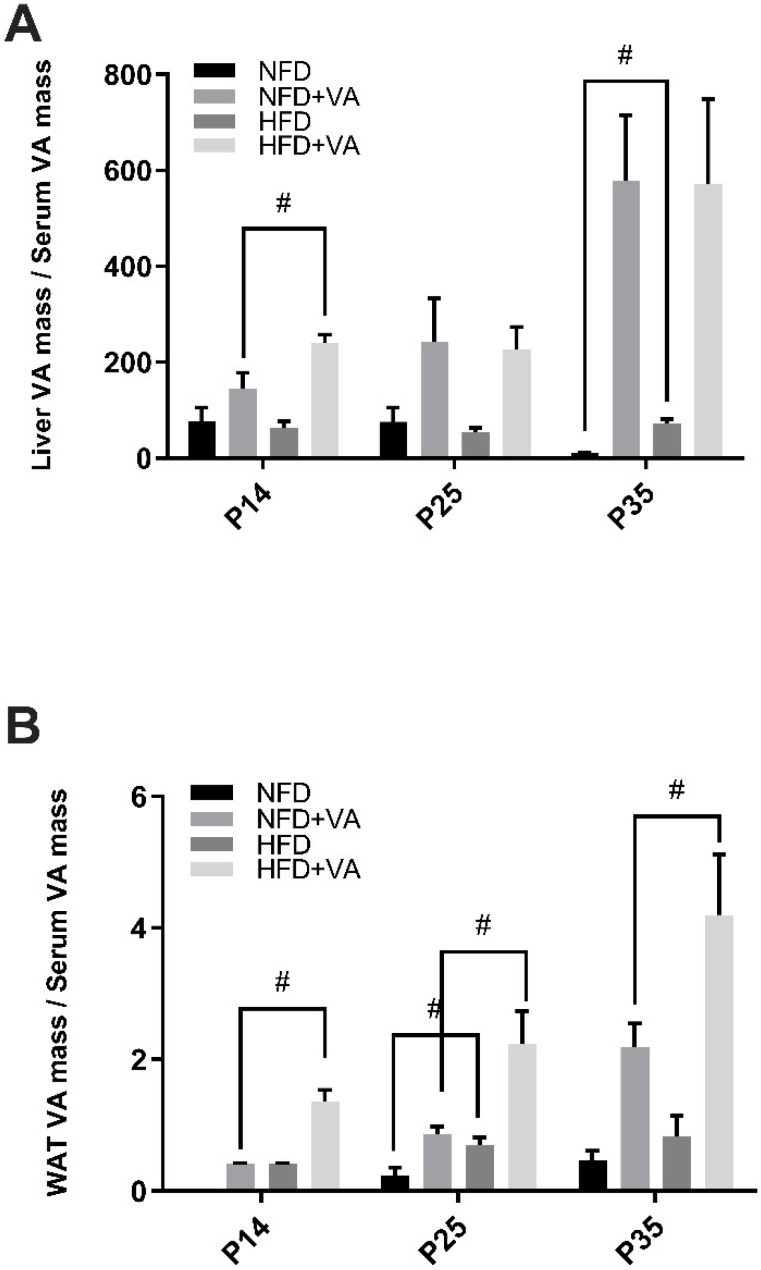
Ratio of total retinol mass in the liver to in serum (**A**), and that in white adipose tissue to in serum (**B**). Bars show mean ± SEM, *n* = 4 or 3 per group. Significant difference, *p* < 0.05, between NFD and HFD or between NFD+VA and HFD+VA by #. P14, postnatal day 14; P25, postnatal day 25; P35, postnatal day 35; NFD, normal fat diet; HFD, high fat diet; VA, vitamin A. Note: At P14, white adipose tissue samples in the NFD group were not adequate for analysis, and therefore data are missing.

The ratio of the total retinol content in the liver or in WAT to that in serum was calculated to demonstrate the relative distribution of VA in its major storage organs versus in circulation (Figure 4). Comparison was conducted between NFD and HFD, and between NFD+VA and HFD+VA. Serum total retinol mass was obtained from the serum total retinol concentration and the estimated serum volume, which is 3.5% of pups’ body weight [36]. As shown, the liver is the major site for VA deposition regardless of the maternal diet. Comparing the NFD and HFD group, a significantly higher ratio of the liver to serum VA mass was noted in the HFD group at P35 (*p* < 0.01) and that of WAT to serum VA mass at P25 (*p* < 0.05). Comparing the NFD+VA and HFD+VA group, a significantly higher ratio of the liver to serum VA mass was observed in the HFD+VA group at P14 and that of WAT to serum VA mass at all three time points (*p* < 0.05).

## 4. Discussion

The current study, to the authors’ knowledge, is the first one to shed light on the potential impact of maternal obesity on the deposition of VA from a maternal VA-adequate or -supplemented diet in neonatal and weanling offspring.

First, as expected from previous findings [30,37], regardless of being added to the NFD or the HFD, maternal VA supplementation during lactation significantly increased the VA concentration in maternal milk and raised the VA status of neonatal rats as evidenced by improved serum and liver VA concentrations. The 14-day-old pups in non-VA-supplemented groups showed a marginal to normal serum VA status and a deficient to marginal liver VA status. The finding is consistent with the well-known fact, as discussed in the introduction section, that newborns have lower VA levels than adults even when their mothers consume adequate VA as in the current study. As pups in the non-supplemented groups grew older, their VA status rose to being normal as indicated by their serum VA concentrations at P25 and P35, confirming the process of VA accumulation upon an adequate supply during growth, which was also reported in human infants [38]. Maternal VA supplementation to both the NFD and the HFD raised the serum VA status to being normal and even being elevated throughout the study. In addition, at P35, the liver VA concentration of VA-supplemented pups indicated a VA sub-toxic status. Rats were inspected on a daily basis for any abnormal symptom by investigators and no typical VA toxicity symptom, including bleeding from the nose, depressed growth, or partial paralysis of the legs, was observed in any rat mother or rat pup. In a study by Felipe et al. [28], an even higher level of dietary VA supplementation (320 mg/kg) was used and toxicity was not noted. However, cautions should still be taken to avoid VA toxicity if maternal VA supplementation is applied to prevent or ameliorate overweight or obesity in infants. In particular, VA supplementation during pregnancy should be considered with extreme caution due to the teratogenic effect of VA.

Secondly, comparing the NFD and the HFD group, a few differences were observed in the liver and adipose tissue VA deposition at P35. Specifically, despite no significant difference in VA intake, the HFD group showed a significantly higher VA concentration and a 10-fold higher VA mass in the liver, as well as a significantly higher ratio of VA mass in the liver to that in serum. These altogether implied more hepatic storage of VA with higher adiposity. VA is stored in hepatic stellate cells (HSCs) as retinyl esters [19]. The increased hepatic level of VA in the HFD group may result from a greater number of HSCs or impaired mobilization of VA from the liver. A recent study reported hypertrophy of HSCs in obese patients with liver fibrosis [39]. In the same study, a specific population of HSCs was observed to present hypertrophy of retinoid droplets in a diet-induced obese murine model with liver fibrosis. Saeed et al. pointed out that various liver diseases, including those associated with obesity and characterized by overactivation and hypertrophy of HSCs, may cause VA metabolic disorders [40]. As such, the long-term impact of maternal obesity on the development of HSCs in the offspring and its potential disturbance on VA metabolism warrants further investigation.

At P35, the HFD group also had a significantly higher VA concentration and mass in their BAT than the NFD group. In addition, at P25, the WAT of HFD pups possessed significantly more VA than the NFD group. However, this may be solely due to the increased tissue weight, since no difference in the VA concentration was noted. Despite these differences in VA deposition, no significant difference in the serum VA concentration was noted between the two groups at any time point. It is postulated that the extent of VA sequestration by the liver as well as by the BAT in HFD pups is not pronounced enough to affect the circulating VA level, which is known to be under tight homeostatic control. Additionally, since pups in these two groups received adequate but not supplemented VA, VA may be mostly delivered to target organs for utilization, and therefore the impact of various factors on VA deposition is limited. In addition, no significant difference in hepatic VA deposition between these two groups was noted at P14 or P25. This indicated that during the short suckling period in rats, the effects of maternal obesity on the neonatal VA status may also be limited, especially when mothers consume adequate VA.

Thirdly, although limited differences in the VA status were found between the NFD and HFD group, especially during the suckling period, the NFD+VA and HFD+VA group displayed more significant differences. Specifically, at P14, as hypothesized, the serum VA concentration was significantly lower in HFD+VA pups; at P25, the VA concentration as well as the total content in the liver, WAT, and BAT were all significantly higher in the HFD+VA group. At P35, the VA contents in both the liver and WAT were significantly higher in the HFD+VA group, potentially owing to the significantly higher tissue weight. In addition, HFD+VA pups exhibited a significantly higher ratio of VA mass in the liver to that in serum at P14 as well as WAT to serum at all three time points. It is unknown why the differences in circulating VA and in storage VA between the two groups were observed at different time points. Previous studies reported an extensive recycling of VA between the plasma and tissues in neonatal rats [41,42]. Therefore, it is postulated that when the liver or adipose tissue harbors more VA as observed at P25 and P35, more retinol is secreted into the circulation for recycling, which offsets the “sink effect” of liver or adipose tissue on serum VA and leads to no difference in the serum VA concentration at these two time points. However, the overall results still imply that when rat mothers consumed supplemented VA, maternal obesity resulted in more VA sequestered in enlarged adipose tissue or liver in offspring, therefore potentially compromising the level of circulating VA that is readily taken up and utilized by other organs. This should be taken into account when VA supplementation is administered to overweight and obese pregnant or lactating women, especially those that are VA deficient. A higher supplementation level may be required to offset the potential impact of maternal obesity on VA availability in their infants.

Lastly, the current study provided information on the distribution of VA in the circulation, liver, and adipose tissue of neonatal and weanling rats reared by mothers receiving normal or excessive dietary fats with adequate or supplemented VA. It was shown that regardless of the fat content or VA content in the maternal diet, the majority of VA is stored in rat pups’ liver. It was also demonstrated that both WAT and BAT took up and stored some VA, while maternal VA supplementation significantly increased the VA concentration in both types of adipose tissue. As mentioned, VA plays an important role in adipose tissue development, especially in the obesogenic environment. The adipose tissue expresses all the enzymes and proteins responsible for VA metabolism, transport, and transcriptional signaling [43]. Therefore, a considerable amount of VA in the adipose tissue may be transformed to retinoic acid and used locally for functions. Moreover, it was found that the concentration of VA in the BAT is similar to that in the WAT, which is consistent with previous findings [44,45,46]. The present study is the first to show that neonatal BAT can sequester more VA with a higher adiposity or when mothers receive additional VA. These findings imply the potential roles of VA in BAT development and adaptive thermogenesis, which contributes to weight control, as previously reported [23,24,26,47].

Although previous studies have documented the beneficial effects of VA on obesity-related metabolic and developmental conditions in adult or young rodent models [47,48,49], the current study provides novel information on how maternal obesity alters the deposition of VA from a VA-adequate or -supplemented maternal diet in the offspring. Multiple strengths should be noted for this study. First, the inclusion of three time points through the suckling and postweaning periods for tissue collection provided a dynamic picture of the VA status and distribution in the early life stage. Secondly, the maternal diets used in the current study contain either 2.6 mg/kg of VA, which was demonstrated in previous studies to support normal growth and development [23,24], so that the VA status of the NFD and HFD group would mimic that of most pregnant women and healthy infants in the U.S., or 129 mg/kg, which was shown in previous research to beneficially regulate adipose tissue development in obese rats [24,27,50]. The adoption of these two dietary VA levels provided information on how maternal obesity affects VA deposition in offspring under VA-adequate or -supplemented condition, allowing for translation of the findings to various public health or clinical settings. Thirdly, milk samples were obtained from P14 sacrifice and provided valuable information. On the one hand, it was shown that the milk VA concentration was not different between the NFD groups and HFD groups. A previous cross-sectional study indicated a significant correlation between a greater pregestational weight and a lower VA concentration in breast milk [51]. A potential explanation of the discrepancy is the short duration that rat mothers of the HFD cohort in the present study were exposed to excessive dietary fat, which was ~4 weeks when the milk samples were obtained. A longer duration of maternal HFD consumption may be needed to observe any potential alteration in milk composition. On the other hand, given the higher percentage of fat in the HFD, one could speculate that more VA is absorbed in HFD rat mothers and passed to their pups, introducing a confounding factor in the analysis of VA status and deposition. However, the results of milk VA excluded that possibility.

Limitations should also be acknowledged for directing future studies. First, since the present study focused on the status and deposition of VA, only serum, liver, and adipose tissue were harvested. Collecting other neonatal organs, especially where VA plays a significant role, such as the lung, brain, reproductive organs, eye, and kidney, will provide insights on the potential alteration of VA uptake and utilization by maternal obesity. Secondly, the current study focused on outcomes in offspring but not in mothers. Obtaining maternal measures in future studies will allow for the establishment of correlations between maternal and neonatal outcomes. Thirdly, in non-obese neonatal rat models, visceral fat can only be discerned from ~P14. In the present study, only ~0.07 g of visceral WAT was able to be collected from the NFD group at P14, which was not adequate for analysis. Lastly, the analysis of genes or proteins involved in VA metabolism and transcriptional signaling will provide insights on how maternal obesity affects VA metabolism in offspring at the molecular level. For instance, in adult models, a significantly positive correlation between the serum RBP4 concentration and adiposity or body mass index was reported [52]. The effect of maternal obesity on RBP4 expression in newborns and infants should be determined in future studies.

Vitamin A deficiency is highly prevalent in pregnant and lactating women in low-income countries, and it results in high morbidity and mortality among infants [53]. It would be of great significance to evaluate maternal diets with a deficient or marginal level of VA in future studies, so that the VA status of women in these areas will be represented and the impacts of maternal obesity on the already compromised VA status of their newborns and infants can be studied. Such findings will inform public health decisions regarding improving the VA status and survival rates of infants in underdeveloped areas. In addition, it is known that maternal obesity increases the risks of preterm delivery [54]. Poor VA status in preterm newborns contributes to a higher likelihood of developing chronic lung disease, such as bronchopulmonary dysplasia, a major cause of morbidity in preterm infants [9]. As such, determining the impacts of maternal obesity on the VA metabolism and the effects of maternal VA supplementation in this population would be valuable.

## 5. Conclusions

To conclude, when rat mothers received an adequate level of dietary VA, maternal HFD consumption had limited impact on the VA status of their offspring during the suckling period. However, weanling pups that continued the consumption of HFD displayed significantly higher VA deposition in their liver and BAT. When mothers consumed supplemented VA, which significantly raised the neonatal VA status, maternal HFD consumption resulted in more VA accumulation in the storage organs in both neonatal and weanling offspring and potentially compromised the availability of functional VA in circulation. A tracer kinetic study using isotope-labeled VA is warranted to obtain a comprehensive understanding of how maternal obesity alters the whole-body VA kinetics in offspring, including its absorption, trafficking, uptake, mobilization, recycling, and clearance.

## Figures and Tables

**Table 1:** Body weight and weights of the liver, visceral white adipose tissue, and brown adipose tissue of rat pups at postnatal day 14, postnatal day 25, and postnatal day 35.

	P14				P25				P35 ^1^			
	NFD	NFD+VA	HFD	HFD+VA	NFD	NFD+VA	HFD	HFD+VA	NFD	NFD+VA	HFD	HFD+VA
BW (g) ^2^	31.69 ± 1.48 ^b^	34.83 ± 0.41 ^ab^	35.96 ± 0.22 ^a^	32.52 ± 0.37 ^b^	72.06 ± 1.84 ^c’^	78.42 ± 1.36 ^b’c’^	96.03 ± 2.78 ^a’^	86.30 ± 1.08 ^b’^	161.90 ± 7.57 ^a”^	152.07 ± 6.72 ^a”^	184.33 ± 6.85 ^a”^	165.63 ± 12.11 ^a”^
LW (g)	0.95 ± 0.07 ^a^	1.10 ± 0.03 ^a^	1.11 ± 0.02 ^a^	1.07 ± 0.04 ^a^	3.31 ± 0.15 ^b’^	3.55 ± 0.09 ^b’^	4.30 ± 0.15 ^a’^	4.24 ± 0.20 ^a’^	8.54 ± 0.58 ^a”^	8.58 ± 0.45 ^a”^	9.05 ± 0.81 ^a”^	8.71 ± 0.35 ^a”^
WATW (g)	0.07 ± 0.02 ^c^	0.15 ± 0.02 ^bc^	0.55 ± 0.04 ^a^	0.25 ± 0.05 ^b^	0.86 ± 0.09 ^c’^	1.20 ± 0.08 ^c’^	3.03 ± 0.11 ^a’^	1.94 ± 0.29 ^b’^	2.69 ± 0.27 ^b”c”^	2.48 ± 0.11 ^c”^	5.45 ± 0.31 ^a”^	3.72 ± 0.27 ^b”^
BATW (g)	0.18 ± 0.02 ^a^	0.18 ± 0.01 ^a^	0.15 ± 0.02 ^a^	0.16 ± 0.03 ^a^	0.35 ± 0.04 ^a’^	0.39 ± 0.02 ^a’^	0.24 ± 0.03 ^b’^	0.50 ± 0.05 ^a’^	0.35 ± 0.04 ^a”^	0.25 ± 0.02 ^a”^	0.39 ± 0.05 ^a”^	0.33 ± 0.02 ^a”^

^1^ Values (*n* = 4 or 3 per group) represent mean ± SEM. Different letters represent significant differences among four groups at individual time point, a > b > c, a’ > b’ > c’, a” > b” > c”, *p* < 0.05. ^2^ BW, body weight; LW, liver weight; WATW, visceral white adipose tissue weight; BATW, brown adipose tissue weight.
